# Complete blood count-derived inflammatory indices as potential markers in threatened abortion: A retrospective cohort study

**DOI:** 10.1097/MD.0000000000051475

**Published:** 2026-11-06

**Authors:** Huseyin Karakaya, Mikail Kusdogan, Gokhan Dogukan Akarsu, Rukiye Hobek Akarsu, Ethem Serdar Yalvac

**Affiliations:** a Department of Obstetrics and Gynecology, Faculty of Medicine, Yozgat Bozok University, Yozgat, Turkey; b Department of Emergency Medicine, Faculty of Medicine, Yozgat Bozok University, Yozgat, Turkey; c Department of Pharmacy Services, Vocational School of Health Services, Yozgat Bozok University, Yozgat, Turkey; d Department of Nursing, Faculty of Health Sciences, Yozgat Bozok University, Yozgat, Turkey.

**Keywords:** complete blood count, Good Health and Well-being (SDG 3), inflammation, lymphocyte, neutrophil-to-lymphocyte ratio, platelet-to-lymphocyte ratio, threatened abortion

## Abstract

This study aimed to evaluate complete blood count-derived inflammatory markers in women with threatened abortion and to investigate their association with threatened abortion. This retrospective cohort study included 802 pregnant women presenting before 20 weeks of gestation. Hematological parameters were compared between women with threatened abortion and healthy pregnant controls. Neutrophil, lymphocyte, platelet, neutrophil-to-lymphocyte ratio (NLR), and platelet-to-lymphocyte ratio (PLR) values were evaluated. Multivariable binary logistic regression analyses were performed to identify factors independently associated with threatened abortion, with primary hematological parameters and derived inflammatory indices assessed in separate models to reduce multicollinearity. Women with threatened abortion had significantly lower lymphocyte counts and higher NLR and PLR values than controls (all *P* < .001), whereas neutrophil and platelet counts did not differ significantly. In multivariable analysis, lower lymphocyte count and higher neutrophil count were independently associated with threatened abortion. In the model including inflammatory indices, both NLR (odds ratio = 2.017, 95% confidence interval = 1.450–2.805; *P* < .001) and PLR (odds ratio = 1.033, 95% confidence interval = 1.022–1.044; *P* < .001) were independently associated with threatened abortion. Threatened abortion was characterized by lower lymphocyte counts and higher NLR and PLR values. These readily available inflammatory indices may serve as useful adjunctive markers for clinical assessment; however, prospective multicenter studies are needed to confirm their predictive utility.

## 1. Introduction

Threatened abortion, also referred to as threatened miscarriage, is one of the most frequently encountered complications during early pregnancy. It is generally characterized by vaginal bleeding before the 20th week of gestation in the presence of a viable intrauterine pregnancy and a closed cervix. Although bleeding may resolve without further complications in a considerable proportion of women, threatened abortion remains clinically important because it is associated with an increased risk of subsequent pregnancy loss and may also be related to adverse obstetric outcomes later in pregnancy. Given that approximately 20% to 25% of clinically recognized pregnancies may be affected, identifying simple parameters that could contribute to the assessment of these patients is of considerable clinical interest.^[1–3]^

The mechanisms underlying threatened abortion have not been fully clarified and are likely to involve several interacting maternal, placental, and immunological factors. Abnormal trophoblast invasion, impaired uteroplacental perfusion, oxidative stress, and alterations in the maternal immune response have all been implicated in early pregnancy complications.^[4–6]^ Inflammation is also thought to play an important role in this process. Pregnancy requires a carefully regulated balance between pro-inflammatory and anti-inflammatory responses, and disruption of this balance may affect implantation, placental development, and maintenance of pregnancy. Therefore, changes in circulating inflammatory cells may provide indirect information regarding the systemic inflammatory and immune alterations accompanying threatened abortion.

In recent years, parameters obtained from routine complete blood count (CBC) analysis have received increasing attention as inexpensive and readily available markers of systemic inflammation. In addition to absolute neutrophil, lymphocyte, and platelet counts, ratios derived from these parameters may provide additional information about the balance between different components of the inflammatory response. The neutrophil-to-lymphocyte ratio (NLR), for example, combines changes in neutrophil-mediated inflammatory activity and lymphocyte-related immune regulation within a single index. Similarly, the platelet-to-lymphocyte ratio (PLR) reflects the relationship between platelet activation and lymphocyte-mediated immune responses. Both indices have been investigated in cardiovascular diseases, malignancies, metabolic disorders, inflammatory conditions, and several obstetric and gynecological settings because they can be calculated easily from routinely available laboratory data.^[7–9]^

The possible role of these indices in pregnancy loss has also been investigated; however, the available findings are not entirely consistent. Bas et al reported that NLR may have a positive predictive role in spontaneous abortion, whereas PLR showed a different pattern of association.^[10]^ Conversely, Liu et al reported no significant predictive value for NLR or PLR in missed miscarriage.^[11]^ Other studies, however, have identified NLR and/or PLR as potentially useful markers of early pregnancy loss.^[12,13]^ Such differences may be related to variations in study design, gestational age at assessment, definitions of pregnancy loss, sample size, and the clinical characteristics of the populations investigated. Importantly, many previous studies have focused on completed or recurrent pregnancy loss rather than on threatened abortion, in which the pregnancy is still viable at the time of clinical presentation.

This distinction is clinically relevant because threatened abortion represents an earlier stage at which the eventual course of pregnancy has not yet been determined. Despite the potential relationship between systemic inflammation and early pregnancy complications, relatively limited data are available regarding CBC-derived inflammatory indices specifically in women presenting with threatened abortion. Therefore, the present study aimed to compare neutrophil, lymphocyte, and platelet counts, together with NLR and PLR, between women with threatened abortion and healthy pregnant controls and to evaluate the independent associations of these hematological parameters with threatened abortion.

## 2. Materials and methods

### 2.1. Study design and setting

This retrospective cohort study was conducted using the medical records of pregnant women who presented to the Department of Obstetrics and Gynecology and the Emergency Department of Yozgat Bozok University Faculty of Medicine between January 2020 and June 2025. Patients were classified according to whether they had been diagnosed with threatened abortion at the time of presentation, and their clinical and laboratory findings were compared with those of pregnant women without threatened abortion.

The inclusion criteria were maternal age between 18 and 45 years, gestational age within the first 20 weeks of pregnancy, presentation to the study hospital during the specified study period, and availability of the demographic, obstetric, clinical, and laboratory data required for the study. The exclusion criteria included maternal age outside the specified range and the presence of medical conditions potentially affecting hematological or inflammatory parameters, including hypothyroidism, renal dysfunction, diabetes mellitus, psoriasis, anemia requiring blood transfusion, and hypertension.

A total of 1124 patient records were retrospectively screened through the hospital’s electronic medical record system. Of these, 322 patients were excluded due to age above 45 years (n = 25), hypothyroidism (n = 68), renal dysfunction (n = 24), diabetes mellitus (n = 128), psoriasis (n = 12), anemia requiring blood transfusion (n = 32), or hypertension (n = 33). Each excluded patient was assigned a single documented reason for exclusion, and no additional exclusion reasons were recorded. Following the application of the eligibility criteria, 802 pregnant women were included in the final study population, comprising 85 women with threatened abortion and 717 controls. Patients were identified retrospectively from available hospital records rather than through consecutive recruitment.

### 2.2. Study population and data collection

Demographic and obstetric characteristics recorded for each participant included maternal age, gravidity, parity, number of previous abortions, and history of abortion.

Information regarding pregnancy outcomes was retrospectively retrieved from the hospital’s electronic medical record system. Pregnancy outcomes were categorized as spontaneous vaginal delivery, cesarean delivery, or dilatation and curettage. Pregnancy outcome analyses were restricted to participants with available outcome data, and percentages were calculated separately for each group using the number of participants with available outcome data as the denominator. Participants with unavailable pregnancy outcome data were excluded only from pregnancy outcome analyses and were retained in other analyses for which the required data were available.

For the analysis of previous abortion history, women with 1 or more previous abortions were categorized as having a positive history of abortion, whereas women with no previous abortion were categorized as having no history of abortion.

### 2.3. Hematological parameters

CBC parameters obtained from routine laboratory testing at the initial clinical presentation were retrieved from the electronic medical records. The hematological parameters evaluated in the study included neutrophil count, lymphocyte count, and platelet count. In addition to these primary hematological parameters, the NLR and PLR were evaluated as CBC-derived inflammatory indices.

These parameters were compared according to selected obstetric characteristics, including gravidity, parity, previous abortion history, and the presence or absence of threatened abortion. In addition, correlations among neutrophil, lymphocyte, platelet, NLR, and PLR values were examined in women presenting with threatened abortion.

### 2.4. Statistical analysis

Statistical analyses were performed using IBM SPSS Statistics version 25.0 (IBM Corp.). Descriptive statistics were expressed as frequencies and percentages for categorical variables and as means and standard deviations for continuous variables. The distribution of continuous variables was evaluated using the Shapiro–Wilk test together with skewness and kurtosis coefficients.

For comparisons between 2 independent groups, the independent-samples *t* test was used. Comparisons involving more than 2 groups were performed using one-way analysis of variance (ANOVA). Categorical variables were compared using the chi-square test or Fisher’s exact test, as appropriate. The distribution of pregnancy outcomes between groups was compared using the Fisher–Freeman–Halton exact test with a Monte Carlo approach. When a statistically significant overall difference was identified, Bonferroni or Games–Howell post hoc tests were applied as appropriate. Pearson correlation analysis was used to examine relationships among the hematological parameters and CBC-derived inflammatory indices. All statistical tests were two-sided, and a *P* value of <.05 was considered statistically significant.

Because the presence of threatened abortion was a binary clinical outcome, multivariable binary logistic regression analysis was used to identify hematological factors independently associated with threatened abortion. To avoid introducing unnecessary collinearity between primary blood cell counts and inflammatory indices mathematically derived from those same parameters, 2 separate regression models were constructed. Model 1 included neutrophil, lymphocyte, and platelet counts together with previous abortion history. Model 2 included NLR and PLR together with previous abortion history. Multicollinearity was assessed using VIFs. The results of the regression analyses were expressed as odds ratios (ORs) with corresponding 95% confidence intervals (CIs). Multivariable logistic regression analyses were performed using a complete-case approach. Of the 802 participants included in the study, 91 were excluded from the regression analyses because of missing data for 1 or more variables required for these models. Consequently, the regression analyses were conducted using data from 711 participants with complete information on the relevant variables.

### 2.5. Ethical considerations

The study was approved by the Yozgat Bozok University Non-Interventional Research Ethics Committee before data collection (Approval No. 2025-GOKAEK-2519_2025.05.11_682; approval date: November 5, 2025). The study was conducted in accordance with the principles of the Declaration of Helsinki, and patient confidentiality was maintained throughout the study. Because of the retrospective nature of the study and the use of previously recorded clinical data, individual informed consent was not required. Institutional permission for the use of the relevant medical records was obtained.

## 3. Results

A total of 802 pregnant women were included in the study. Of these, 85 women were diagnosed with threatened abortion, while 717 women constituted the control group. The mean maternal age was 29.33 ± 5.39 years. Most participants were multigravida (98.0%), and 71.2% were nulliparous. A previous abortion history was present in 170 women (21.2%), whereas 632 women (78.8%) had no previous abortion (Table 1).

**Table 1:** Distribution of participants’ sociodemographic and obstetric characteristics.

Characteristics	n	%
Mean age (yr)	29.33 ± 5.39	
Gravidity		
Primigravida	16	2.0
Multigravida	786	98.0
Mean gravidity	2.13 ± 1.44	
Parity		
0	571	71.2
1	138	17.2
2	65	8.1
≥3	28	3.5
Mean parity	0.44 ± 0.79	
History of abortion		
Yes	170	21.2
None	632	78.8
Mean abortion	0.30 ± 0.72	

Pregnancy outcome data were available for 445 (55.5%) of the 802 pregnant women included in the study. Of these, 60 women belonged to the threatened abortion group, representing 70.6% of this group, while 385 women belonged to the control group, representing 53.7% of controls. Among women with threatened abortion and available pregnancy outcome data, 32 (53.3%) underwent cesarean delivery, 22 (36.7%) had a spontaneous vaginal delivery, and 6 (10.0%) underwent dilatation and curettage. In the control group, 222 (57.7%) women underwent cesarean delivery and 163 (42.3%) had a spontaneous vaginal delivery, whereas no dilatation and curettage cases were recorded. The overall distribution of pregnancy outcomes differed significantly between the 2 groups (*P* < .001), primarily due to the occurrence of dilatation and curettage exclusively in the threatened abortion group (Table 2).

**Table 2:** Comparison of pregnancy outcomes according to threatened abortion status.

Characteristics	Threatened abortion (n = 85)	Control (n = 717)	Total (n = 802)	*P* value
Pregnancy outcome availability, n (%)				
Available)	60 (70.6)	385 (53.7)	445 (55.5)	
Unavailable	25 (29.4)	332 (46.3)	357 (44.5)	
Pregnancy outcomes, n (%)				<.001
Cesarean section	32 (53.3)	222 (57.7)	254 (57.1)	
Spontaneous vaginal delivery	22 (36.7)	163 (42.3)	185 (41.6)	
Dilatation and curettage	6 (10.0)	0 (0.0)	6 (1.3)	

Pregnancy outcome percentages were calculated using participants with available outcome data in each group (n = 60 and n = 385, respectively). Percentages for outcome data availability were calculated using the total number of participants in each group (n = 85 and n = 717, respectively). The overall distribution of pregnancy outcomes was compared using the Fisher–Freeman–Halton exact test with a Monte Carlo approach.

Comparison of demographic and obstetric characteristics between women with threatened abortion and healthy pregnant controls revealed significant differences in maternal age, parity distribution, and previous abortion history. Women with threatened abortion were significantly older than controls (31.54 ± 5.87 vs 29.07 ± 5.28 years; *P* < .001). Mean parity was significantly higher in women with threatened abortion than in controls (1.08 ± 0.92 vs 0.36 ± 0.73; *P* < .001). Similarly, the mean number of previous abortions was significantly higher in the threatened abortion group (0.48 ± 0.83 vs 0.27 ± 0.71; *P* = .028; Table 3).

**Table 3:** Comparison of demographic and obstetric characteristics between women with and without threatened abortion.

Characteristics	Threatened abortion (n = 85)	Control (n = 717)	*P* value
Maternal age (yr), mean ± SD	31.54 ± 5.87	29.07 ± 5.28	<.001
Gravidity, mean ± SD	2.42 ± 1.45	2.10 ± 1.44	.055
Gravidity, n (%)			.397
Primigravida	0 (0.0)	16 (2.2)	
Multigravida	85 (100.0)	701 (97.8)	
Parity, mean ± SD	1.08 ± 0.92	0.36 ± 0.73	<.001
Parity, n (%)			<.001
0	24 (28.2)	547 (76.3)	
1	38 (44.7)	100 (13.9)	
2	15 (17.6)	50 (7.0)	
≥3	8 (9.4)	20 (2.8)	
Previous abortions, mean ± SD	0.48 ± 0.83	0.27 ± 0.71	.028
History of abortion, n (%)			.002
Yes	30 (35.3)	140 (19.5)	
No	55 (64.7)	577 (80.5)	

SD = standard deviation.

Furthermore, a history of previous abortion was significantly more common in the threatened abortion group than in the control group (35.3% vs 19.5%, *P* = .002). Although mean gravidity was higher in women with threatened abortion, this difference did not reach statistical significance (*P* = .055). When hematological parameters were evaluated according to gravidity, no statistically significant differences were observed in neutrophil, lymphocyte, platelet, NLR, or PLR values between primigravid and multigravid women (all *P* > .05). In contrast, parity was significantly associated with lymphocyte count, NLR, and PLR (all *P* < .001), whereas neutrophil and platelet counts did not differ significantly across parity groups. Post hoc comparisons showed that nulliparous women had higher lymphocyte counts than women with parity of 1 or 2, while NLR and PLR differed mainly between nulliparous women and those with parity of 1 (Table 4).

**Table 4:** Comparison of hematological parameters according to selected obstetric characteristics.

Characteristics	Hematological profiles of participants				
	Neutrophils	Lymphocytes	Platelets	NLR	PLR
	x̄ ± SD	x̄ ± SD	x̄ ± SD	x̄ ± SD	x̄ ± SD
Gravidity					
Primigravida	7.17 ± 3.46	9.99 ± 3.77	273.08 ± 76.96	0.76 ± 0.38	29.68 ± 11.09
Multigravida	7.03 ± 4.45	8.72 ± 3.65	251.95 ± 84.88	1.15 ± 1.59	42.44 ± 60.22
Test*	0.120	1.243	0.857	−1.051	−0.928
*P* value	.904	.214	.392	.293	.354
Parity					
0 (a)	7.02 ± 3.98	9.33 ± 3.34	253.14 ± 90.67	0.94 ± 1.41	36.07 ± 61.35
1 (b)	7.28 ± 6.45	7.04 ± 3.79	253.31 ± 71.32	1.74 ± 2.04	59.38 ± 54.95
2 (c)	6.39 ± 3.03	7.73 ± 4.28	246.68 ± 65.66	1.30 ± 1.37	52.51 ± 46.79
≥3 (d)	7.38 ± 2.46	7.84 ± 4.30	242.96 ± 62.36	1.66 ± 1.77	56.40 ± 58.47
Test†	0.628	17.639	0.225	13.066	17.548
*P* value	.597	<.001	.879	<.001	<.001
Identification of intergroup differences	–	a > b,c	–	a < b	a < b
History of abortion					
Yes	6.60 ± 2.45	8.13 ± 3.84	248.82 ± 68.02	1.26 ± 1.54	48.26 ± 50.50
No	7.15 ± 4.84	8.92 ± 3.59	253.23 ± 88.84	1.11 ± 1.59	40.58 ± 62.03
Test*	−1.391	−2.463	−0.592	1.500	2.194
*P* value	.165	.014	.554	.135	.029
Threatened abortion					
Yes	7.02 ± 2.78	2.41 ± 2.45	259.94 ± 76.06	3.85 ± 2.37	138.19 ± 61.07
No	7.03 ± 4.60	9.53 ± 2.95	251.32 ± 85.78	0.78 ± 0.98	30.22 ± 47.55
Test*	−0.019	−24.666	0.884	12.048	16.515
*P* value	.985	<.001	.377	<.001	<.001

ANOVA = analysis of variance, NLR = neutrophil-to-lymphocyte ratio, PLR = platelet-to-lymphocyte ratio, SD = standard deviation.

* Independent samples *t* test.

† One-way ANOVA.

Women with a previous history of abortion had significantly lower lymphocyte counts than women without such a history (*P* = .014). PLR values were significantly higher in women with a previous abortion history (*P* = .029). No statistically significant differences were observed between the groups in neutrophil count, platelet count, or NLR (all *P* > .05; Table 4).

The most pronounced hematological differences were observed according to threatened abortion status. Women presenting with threatened abortion had markedly lower lymphocyte counts than healthy pregnant controls (2.41 ± 2.45 vs 9.53 ± 2.95; *P* < .001). In contrast, both NLR and PLR were significantly higher in the threatened abortion group (3.85 ± 2.37 vs 0.78 ± 0.98 for NLR and 138.19 ± 61.07 vs 30.22 ± 47.55 for PLR; both *P* < .001). Neutrophil and platelet counts did not differ significantly between the groups (*P* > .05; Table 4).

Following the comparison of hematological parameters between the study and control groups, correlation analysis was conducted specifically within the threatened abortion group. A moderate positive correlation was observed between neutrophil count and NLR (*r* = 0.590, *P* < .001). Lymphocyte count was moderately and negatively correlated with both NLR (*r* = −0.396, *P* < .001) and PLR (*r* = −0.496, *P* < .001). Platelet count showed a moderate negative correlation with NLR (*r* = −0.367, *P* = .001) and a weak positive correlation with PLR (*r* = 0.226, *P* = .038). NLR and PLR were also moderately positively correlated with each other (*r* = 0.484, *P* < .001; Table 5).

**Table 5:** Correlation matrix of hematological parameters in women presenting with threatened abortion.

		Neutrophil	Lymphocyte	Platelet	NLR	PLR
Neutrophil	*r*= *P*=	–	−0.003 (.980)	−0.157 (.151)	**0.590** **(<.001**)	−0.202 (.064)
Lymphocyte	*r*= *P*=	−0.003 (.980)	–	0.054 (.623)	**−0.396** **(<.001**)	**−0.496 (<.001**)
Platelet	*r*= *P*=	−0.157 (0.151)	0.054 (623)	–	**−0.367** **(.001**)	0.226 (.038)
NLR	*r*= *P*=	**0.590** **(<.001**)	**−0.396** **(<0.001**)	**−0.367** **(.001**)	–	**0.484** **(<.001**)
PLR	*r*= *P*=	−0.202 (.064)	**−0.496** **(<.001**)	0.226 (.038)	**0.484** **(<.001**)	–

Values are presented as Pearson’s correlation coefficient (*r*), with *P* values in parentheses.

Bold values indicate statistical significance at the *P* < .05 level.

NLR = neutrophil-to-lymphocyte ratio, PLR = platelet-to-lymphocyte ratio.

Values are presented as Pearson’s correlation coefficient (*r*), with *P* values in parentheses.

Multivariable binary logistic regression analyses were then performed to identify factors independently associated with threatened abortion. In the model including the primary hematological parameters, a higher neutrophil count (OR = 1.093, 95% CI = 1.038–1.150; *P* < .001) and a lower lymphocyte count (OR = 0.291, 95% CI = 0.228–0.371; *P* < .001) were independently associated with threatened abortion. Platelet count and previous abortion history were not independently associated with threatened abortion in this model.

In the second model, which included CBC-derived inflammatory indices, both NLR and PLR remained independently associated with threatened abortion. A higher NLR was associated with increased odds of threatened abortion (OR = 2.017, 95% CI = 1.450–2.805; *P* < .001), as was a higher PLR (OR = 1.033, 95% CI = 1.022–1.044; *P* < .001). Previous abortion history did not reach statistical significance in this model (*P* = .069; Table 6).

**Table 6:** Multivariable binary logistic regression analysis of factors associated with threatened abortion.

Model	Predictor	OR	95% CI	*P* value
Model 1: primary hematological parameters	Neutrophil	**1.093**	**1.038–1.150**	**<.001**
	Lymphocyte	**0.291**	**0.228–0.371**	**<.001**
	Platelet	1.006	0.999–1.013	.099
	Previous abortion history	1.839	0.592–5.714	.293
Model 2: CBC-derived inflammatory indices	NLR	**2.017**	**1.450–2.805**	**<.001**
	PLR	**1.033**	**1.022–1.044**	**<.001**
	Previous abortion history	2.093	0.944–4.643	.069

Binary logistic regression was performed with threatened abortion status as the dependent variable.

Bold values indicate statistical significance at the *P* < .05 level.

CBC = complete blood count, CI = confidence interval, NLR = neutrophil-to-lymphocyte ratio, OR = odds ratio, PLR = platelet-to-lymphocyte ratio.

*Binary logistic regression was performed with threatened abortion status as the dependent variable.

ORs and 95% CIs are reported. Primary hematological parameters and CBC-derived inflammatory indices were evaluated in separate models to reduce potential multicollinearity arising from the mathematical derivation of NLR and PLR from the underlying cell counts. Of the 802 participants included in the study, 91 (11.3%) were excluded from the multivariable logistic regression analyses due to missing data for 1 or more variables required for these models. Consequently, the regression analyses were performed using a complete-case approach involving 711 participants (88.7%) with complete data for the relevant variables.

## 4. Discussion

The present study evaluated routine hematological parameters and CBC-derived inflammatory indices in a relatively large cohort of pregnant women with and without threatened abortion. The main findings were that women presenting with threatened abortion had markedly lower lymphocyte counts and higher NLR and PLR values than healthy pregnant controls, whereas neutrophil and platelet counts alone did not differ significantly between the groups. In addition, multivariable analyses showed that a lower lymphocyte count and a higher neutrophil count were independently associated with threatened abortion when primary hematological parameters were considered, while both NLR and PLR remained independently associated with threatened abortion in the model based on CBC-derived inflammatory indices. These findings suggest that the relative distribution of circulating inflammatory cells may provide more information than individual cell counts alone in women presenting with threatened abortion.

Inflammatory and immunological adaptations are essential for implantation, placentation, and the maintenance of pregnancy. Although pregnancy is accompanied by physiological changes in circulating leukocyte populations, excessive or dysregulated inflammatory activity has been implicated in several adverse pregnancy outcomes. Changes in neutrophil and lymphocyte populations may therefore reflect alterations in the balance between innate and adaptive immune responses. NLR integrates these 2 cellular components and has increasingly been investigated as a simple indicator of systemic inflammatory activity. PLR similarly combines platelet-related inflammatory and thrombotic activity with lymphocyte-mediated immune responses. Because both indices can be calculated from routine CBC measurements without additional laboratory testing or cost, they have attracted interest as potential adjunctive markers in reproductive and obstetric disorders.

Previous studies examining pregnancy loss have produced heterogeneous results. Bas et al reported an association between CBC-derived inflammatory markers and spontaneous abortion, suggesting a potential role for NLR in identifying women at increased risk of pregnancy loss.^[10]^ In a case-control study, Yazdizadeh et al also evaluated NLR and PLR during the first trimester and reported differences in inflammatory indices between women with spontaneous miscarriage and controls.^[13]^ Conversely, other investigations have failed to demonstrate consistent discriminatory or prognostic value for these parameters. Such discrepancies are not unexpected, because studies differ considerably in terms of gestational age, definition of pregnancy loss, timing of blood sampling, exclusion of inflammatory comorbidities, and whether the population consists of women with threatened, missed, recurrent, or completed miscarriage.

Our findings regarding threatened abortion are particularly notable for the pronounced reduction in lymphocyte count accompanied by elevations in NLR and PLR. In contrast, neutrophil and platelet counts considered individually were not significantly different between women with threatened abortion and healthy controls. This pattern may indicate that the relative balance between circulating cell populations is more informative than isolated cell counts. Ata et al similarly investigated NLR and PLR in women with threatened abortion and early pregnancy loss, while Isenlik et al evaluated serum and hematological parameters in threatened miscarriage.^[14,15]^ Taken together with the present findings, these studies support continued investigation of readily available hematological indices in early-pregnancy complications, although differences across populations prevent the establishment of universal reference values or clinical thresholds at present.

The association between lymphocyte count and threatened abortion deserves particular attention. In the present cohort, lymphocyte levels were substantially lower in women with threatened abortion than in controls, and lower lymphocyte count remained independently associated with threatened abortion in the multivariable model. This finding may reflect altered maternal immune regulation during an unstable early pregnancy. However, lymphocyte count is influenced by a wide range of physiological and clinical factors, and a single measurement cannot establish the underlying immunological mechanism. For this reason, the lymphocyte findings should be interpreted together with the derived indices rather than as evidence that lymphocyte count alone can identify women who will subsequently experience pregnancy loss.

Previous abortion history was also associated with differences in some hematological parameters. Women with a history of abortion had lower lymphocyte counts and higher PLR values than women without such a history. However, previous abortion history did not remain independently associated with threatened abortion in either multivariable model. Similarly, although NLR was numerically higher in women with a previous abortion, the difference was not statistically significant. This distinction is important because recurrent pregnancy loss has previously been associated with inflammatory alterations. Hantoushzadeh et al, in a systematic review and meta-analysis, examined the diagnostic value of NLR and PLR in recurrent pregnancy loss and abortion and demonstrated that inflammatory indices may differ in these populations.^[16]^ The present findings suggest that previous pregnancy loss and the inflammatory profile accompanying a current episode of threatened abortion may be related but should not be considered interchangeable clinical phenomena.

CBC-derived inflammatory indices have also been investigated in other obstetric and gynecological conditions, illustrating both their potential usefulness and their limitations. Aktoz et al assessed hematological inflammatory markers in women hospitalized with tubo-ovarian abscess and found that clinical findings and CRP, rather than hematological indices alone, were more useful in predicting medical treatment success.^[17]^ In another study involving women undergoing cesarean delivery, Aktoz et al evaluated preoperative and postoperative CBC parameters for the prediction of surgical site infection. Although postoperative changes were observed in WBC, neutrophil, lymphocyte, NLR, and PLR values, CBC-derived parameters did not reliably predict subsequent surgical site infection.^[18]^

These observations are relevant to the present study because they demonstrate that alterations in CBC-derived inflammatory indices occur across a range of obstetric and gynecological inflammatory conditions, but their clinical significance depends strongly on the underlying disease process and the outcome being assessed. Accordingly, NLR and PLR should not be viewed as disease-specific biomarkers.

The correlation analysis in the threatened abortion group further supports the interrelated nature of these hematological indices. NLR was positively correlated with neutrophil count and negatively correlated with lymphocyte and platelet counts, whereas PLR showed a negative correlation with lymphocyte count and a positive correlation with platelet count. NLR and PLR were also positively correlated with each other. These relationships are biologically and mathematically plausible because both indices are derived from common cellular components, particularly lymphocyte count. They also reinforce the need to consider potential collinearity when these variables are included in multivariable analyses. For this reason, primary blood cell counts and derived inflammatory indices were evaluated in separate logistic regression models in the present study.

In the multivariable analysis, higher NLR and PLR were independently associated with the presence of threatened abortion. The association was particularly pronounced for NLR, whereas the OR for PLR reflected a smaller change per 1-unit increase in the index. These findings support an association between an altered systemic inflammatory profile and threatened abortion, but they should not be interpreted as demonstrating prospective predictive performance. The hematological measurements were obtained in relation to the clinical presentation, and the regression models evaluated the presence of threatened abortion rather than subsequent miscarriage as the outcome. Therefore, although NLR and PLR may be useful adjunctive parameters in the clinical assessment of these patients, their ability to predict pregnancy loss or other later obstetric outcomes requires dedicated prospective evaluation.

## 5. Limitations

Several limitations should be considered when interpreting these findings. First, the retrospective and single-center design limits the ability to establish temporal or causal relationships and may reduce the generalizability of the results to other populations. Although the overall sample size was relatively large, pregnancy outcome data were unavailable within the study center for 357 women who were followed up or delivered elsewhere. Therefore, the current analyses primarily address the association between hematological parameters and threatened abortion at presentation rather than prediction of the final pregnancy outcome.

Second, CBC parameters were based on measurements obtained at the initial presentation, and serial changes during the course of pregnancy could not be evaluated. Hematological indices may vary with gestational age and may also be influenced by transient inflammatory, physiological, or environmental factors. Furthermore, although patients with known chronic systemic diseases were excluded, residual confounding related to unrecorded clinical factors cannot be completely excluded because of the retrospective nature of the study. The multivariable regression analyses were also based on participants with complete data for the variables included in the respective models.

Finally, NLR and PLR are nonspecific inflammatory indices. They are attractive because they are inexpensive and readily accessible, but they should not be considered substitutes for clinical assessment or more specific inflammatory markers. Future prospective multicenter studies incorporating serial hematological measurements, detailed clinical characteristics, additional inflammatory biomarkers, and complete pregnancy follow-up are needed to determine whether these indices provide clinically meaningful incremental value and to establish appropriate thresholds for risk stratification.

## 6. Conclusion

This study demonstrated that women with threatened abortion had significantly lower lymphocyte counts and higher NLR and PLR values than healthy pregnant controls. In multivariable analyses, lower lymphocyte count and higher neutrophil count were independently associated with threatened abortion, while NLR and PLR remained independently associated with threatened abortion when CBC-derived inflammatory indices were evaluated separately. These findings suggest that NLR and PLR may provide useful, inexpensive, and readily available supplementary information in the clinical assessment of women presenting with threatened abortion. However, because of the retrospective and single-center design of the study, these parameters should not be considered standalone predictive biomarkers. Prospective multicenter studies with complete pregnancy follow-up are needed to determine their predictive performance and clinical applicability.

## Acknowledgments

The authors would like to thank all the participants who participated in this study.

## Author contributions

**Conceptualization:** Huseyin Karakaya, Mikail Kusdogan, Gokhan Dogukan Akarsu, Rukiye Hobek Akarsu, Ethem Serdar Yalvac.

**Data curation:** Huseyin Karakaya, Mikail Kusdogan, Gokhan Dogukan Akarsu, Rukiye Hobek Akarsu, Ethem Serdar Yalvac.

**Formal analysis:** Huseyin Karakaya, Mikail Kusdogan, Gokhan Dogukan Akarsu, Rukiye Hobek Akarsu, Ethem Serdar Yalvac.

**Validation:** Huseyin Karakaya, Mikail Kusdogan, Gokhan Dogukan Akarsu, Rukiye Hobek Akarsu, Ethem Serdar Yalvac.

**Writing – original draft:** Huseyin Karakaya, Mikail Kusdogan, Gokhan Dogukan Akarsu, Rukiye Hobek Akarsu, Ethem Serdar Yalvac.

**Writing – review & editing:** Huseyin Karakaya, Mikail Kusdogan, Gokhan Dogukan Akarsu, Rukiye Hobek Akarsu, Ethem Serdar Yalvac.
